# Detection and quantification of *Verticillium dahliae* and *V. longisporum* by droplet digital PCR versus quantitative real-time PCR

**DOI:** 10.3389/fcimb.2022.995705

**Published:** 2022-08-22

**Authors:** Di Wang, Xinya Jiao, Haijiang Jia, Shumei Cheng, Xi Jin, Youhua Wang, Yunhua Gao, Xiaofeng Su

**Affiliations:** ^1^ Center for Advanced Measurement Science, National Institute of Metrology, Beijing, China; ^2^ College of Food Science and Technology, Hebei Agricultural University, Baoding, China; ^3^ Raw Material Technology Center of Guangxi Tobacco, Nanning, China; ^4^ Hebei Technology Innovation Center for Green Management of Soil-Borne Diseases, Baoding University, Hebei, China; ^5^ Biotechnology Research Institute, Chinese Academy of Agricultural Sciences, Beijing, China

**Keywords:** droplet digital PCR, quantitative real-time PCR, sensitivity, quantification, tolerance

## Abstract

Vascular wilt, caused by *Verticillium dahliae* and *V. longisporum*, limits the quality and yield of agricultural crops. Although quantitative real-time PCR (qPCR) has greatly improved the diagnosis of these two pathogens over traditional, time-consuming isolation methods, the relatively poor detection sensitivity and high measurement bias for traceable matrix-rich samples need to be improved. Here, we thus developed a droplet digital PCR (ddPCR) assay for accurate, sensitive detection and quantification of *V. dahliae* and *V. longisporum*. We compared the analytical and diagnostic performance in detail of ddPCR and the corresponding qPCR assay against the genomic DNA (gDNA) of the two fungi from cultures and field samples. In our study, the species specificity, quantification linearity, analytical sensitivity, and measurement viability of the two methods were analyzed. The results indicated that ddPCR using field samples enhanced diagnostic sensitivity, decreased quantification bias, and indicated less susceptibility to inhibitors compared with qPCR. Although ddPCR was as sensitive as qPCR when using gDNA from cultures of *V. dahliae* and *V. longisporum*, its detection rates using field samples were much higher than those of qPCR, potentially due to the inhibition from residual matrix in the extracts. The results showed that digital PCR is more sensitive and accurate than qPCR for quantifying trace amounts of *V. dahliae* and *V. longisporum* and can facilitate management practices to limit or prevent their prevalence.

## Introduction

Substantial losses in quality and crop yield are caused by soilborne pathogens such as *Verticillium dahliae* and *V. longisporum*, which cause severe vascular wilt diseases characterized by plant stunting, discoloration, wilting, and death, across a broad host range (Isaac, 1946; Xiao et al., 1997). These wilt diseases result in 10–50% yield loss and an annual economic loss of USD 3 billion worldwide (Depotter et al., 2016). No fungicides are effective against these fungi because they are present in the host vasculature and the soil. The preferred and most economically efficient management strategy is to block the transport of *V. dahliae* and *V. longisporum* during the early stage of infection (Anguita-Maeso et al., 2021). Thus, accurate, sensitive quantitative detection methods are needed to assess risk and outbreaks as early as possible.

Traditional methods used to identify *V. dahliae* and *V. longisporum* include isolating the pathogen and observing its morphology, which is laborious, time-consuming, and lacks specificity (Mahanty, 1970). PCR, based on differences in the characteristics between fungal genomic and specific DNA fragments, has been applied to distinguish *Verticillium* species (Robb et al., 1993; Mercado-Blanco et al., 2002; Moradi et al., 2014). Quantitative real-time PCR (qPCR) is superior in comparison with traditional methods in terms of accuracy, specificity, and quantification range; therefore, it is commonly used for monitoring *Verticillium* spp. in plant and environmental samples (Dan et al., 2001; Gayoso et al., 2007; Duressa et al., 2012). However, qPCR assays are generally sensitive to PCR inhibitors in field samples, which may lead to false negative results (Aljawasim and Vincelli, 2015). Moreover, accurate quantification relies on standard curves obtained using serial dilutions of standards, and marked variations in amplification efficiency often result in poor reproducibility and inconsistencies between different laboratories (Bustin et al., 2009; Cao et al., 2013).

Digital PCR (dPCR) is increasing in popularity due to its high sensitivity, increased tolerance to some PCR inhibitors, and improved accuracy and repeatability (Hindson et al., 2013; Pavšič et al., 2016). As a calibration-free method, it directly quantifies the absolute copy number concentration of targets in samples, based only on the frequency of positive droplets and an approximation of Poisson distribution (Pinheiro et al., 2012). Because of these benefits, dPCR has been used for purposes such as reference material development and clinical diagnostics (Pohl and Shih Ie, 2004; Milosevic et al., 2018; Li et al., 2020).

Compared with the abundant studies on qPCR, which is routinely utilized in various applications, studies on digital PCR for plant pathogen detection are limited. Therefore, a systematic comparison of digital PCR and qPCR performance is required. Here, we developed a droplet digital PCR (ddPCR) assay for quantitative detection of *V. dahliae* and *V. longisporum*. We further evaluated the performance of both methods in terms of species specificity, analytical sensitivity, quantification limits, measurement precision, quantitative correlation, and influence of the residual matrix using cultured and field samples.

## Materials and methods

### Fungal, bacterial, soil, and plant samples

The *V. dahliae* strain (highly virulent defoliating V991) was stored in our laboratory. We obtained isolates of 11 species of fungi (*V. longisporum*, *V. nonalfalfae*, *V. alboatrum*, *V. nigrescens*, *Magnaporthe oryzae*, *Bipolaris maydis*, *Exserohilum turcicum*, *Rhizoctonia cerealis*, *Meloidogyne incognita*, *Fusarium pseudograminearum*, and *Fusarium oxysporum* f. sp. *conglutinans* [Foc]) and 6 isolates of bacteria (*Ustilaginoidea virens*, *Acidovorax citrulli*, *Xanthomonas oryzae* pv. *oryzae* [Xoo], *Pseudomonas syringae*, *Ralstonia solanacearum*, and *Xanthomonas campestris* pv. *campestris* [Xcc]) from the Chinese Academy of Agricultural Sciences and China Agricultural University. All samples were stored in 25% glycerol at -80°C.

Fresh cotton roots were collected from an experimental field (Langfang, Hebei Province, China, 39˚51’N, 116˚60’E) and quickly frozen in liquid nitrogen. Soil samples were collected from the surface of cotton roots (10-20 cm), air-dried, then brushed through a 2-mm mesh sieve. We collected 500 mg of field soil and 500 mg of fresh cotton roots for each sample.

### Phylogenetic tree construction

Intergenic spacer (IGS) sequences from *V. dahliae* and *V. longisporum* were downloaded from the National Center for Biotechnology Information (NCBI) database. IGS homologs from the genomes of eight *Verticillium* fungi, including *V. nonalfalfae*, *V. albo-atrum*, *V. alfalfae*, *V. zaregamsianum*, *V. nubilum*, *V. isaacii*, *V. tricorpus*, and *V. klebahnii* were obtained from the NCBI online BLAST tool. A phylogenetic tree was constructed based on the sequences using MEGA 11.0 software with the neighbor-joining method and 1000 bootstrap replications.

### DNA extraction and molecular identification of fungi and bacteria

Biological samples were cultured in complete medium/Luria-Bertani broth at 25/37°C. The cultured samples were harvested at 3000 × *g* for 10 min, and the genomic DNA (gDNA) was extracted using a commercial kit and the instruction manual (KG203, Tiangen, Beijing, China). gDNA of cotton roots was extracted using a Hi-DNAsecure Plant Kit and the instructions (DP350, Tiangen). gDNA of soil samples was extracted using another kit and the provided protocol (DP336; Tiangen). The quality and concentration of gDNA was determined using 1% agarose gel electrophoresis and a NanoDrop Spectrophotometer (ThermoFisher, USA). Then, all samples were aliquoted and stored them in a refrigerator at -80°C as templates to develop the qPCR and ddPCR assays.

For the above fungal and bacterial samples, the internal transcribed spacer (ITS) of the 18S ribosomal DNA (rDNA) was amplified using the specific primer pair 5’-TCCGTAGGTGAACCTGCGG-3’/5’-TCCTCCGCTTATTGATATGC-3’. The 16S ribosomal RNA (rRNA) gene was amplified with the bacterial universal primer pair 27F (5’-AGAGTTTGATCCTGGCTCAG-3’)/1492R (5’-GGTTACCTTGTTACGACTT-3’). The amplicons were sequenced by Sangon Biotech Co., Ltd. (Shanghai, China), and the obtained sequences results were aligned using the Basic Local Alignment Search Tool (https://blast.ncbi.nlm.nih.gov/Blast.cgi) (Wu et al., 2002; Su et al., 2021).

### Evaluated primer/probe sets

Sequences of primer/probe sets for the qPCR and ddPCR experiments were designed based on published references (**Table 1**) and synthesized by Shanghai Sangon Biotech Co., Ltd. (Shanghai, China). The qPCR assays were done using previously reported protocols. Stock solutions of the primers were made at 100 μM and diluted to 10 μM as the final working solution.

**Table 1:** Primers and probes used in this study.

Name	Target	Oligo sequence (5’-3’)	Product length (bp)	Reference
Vdl-1	IGS (AF104926)	F: CGTTTCCCGTTACTCTTCT	159	(Bilodeau et al., 2012)
		R: GGATTTCGGCCCAGAAACT		
		P: FAM CACCGCAAGCAGACTCTTGAAAGCCA BHQ-1		
Vdl-2	β-tubulin (AY354459)	F: CTCGATCGTCGTCAACC	155	(Pasche et al., 2013)
		R: TGGTGGTGAGAGTGTTG		
		P: FAM-TACGACAACGACTTCGCCATC BHQ-1		
Vdl-3	V357I (DQ266246)	F: GGCTCAAGTTAACTACGG	123	(Maurer et al., 2013)
		R: CTGTCATGTATATAAGATACTACTG		
		P: FAM-AGGTATAAGGTCCATATCCAACACGAG BHQ-1		
Vdl-4	VDAG_05595	F: GGCTCAAGTTAACTACGG	104	(Duressa et al., 2013)
		R: TTGGACTTCACATTGTCGATCGT		
		P: FAM-TTGGAAGTCGAATCATCC BHQ-1		
Vdl-5	IGS (AF104926)	F: GGGAAGAGAGAGCGAGAG	113	(Feng et al., 2014)
		R: GTAGGCGGCCGTGACAG		
		P: FAM-GCGCCTTGCTCTAGCGACCT BHQ-1		

### qPCR assay

The qPCRs were done using an ABI 7500 Fast Real-Time PCR system (Applied Biosystems, USA). The 20 μL reaction mixture contained 2×AceQ Universal U and Probe Master Mix V2 (Vazyme Biotech Co., Ltd, Nanjing) (10 μL), forward and reverse primer (each 0.4 μL), probe (0.2 μL), gDNA (2 μL), and nuclease-free water (7 μL). The thermal cycling conditions were denatured at 95°C for 5 min, followed by 40 cycles of denaturation at 95°C for 10 s and annealing and elongation at 60°C for 30 s. Quantification cycle (Qc) values were calculated using 7500 software v2.3 (Applied Biosystems). We used distilled water as the template for the negative control.

The standard curves for qPCR were constructed using 10-fold serial dilutions of *V. dahliae* and *V. longisporum* gDNA as templates, the concentration of which we measured on a Nanodrop (Thermo Fischer Scientific, USA). Three independent experiments were carried out with four reactions of each concentration to generate the standard curves. We plotted the standard curve by the Cq values of the qPCR assays against the corresponding logarithm (base 10) of the concentrations of these dilutions.

### ddPCR assay

The ddPCR assay was performed using ddPCR™ Supermix for Probes (Bio-Rad Laboratories, USA) on a QX200 Droplet Digital PCR System (Bio-Rad Laboratories, USA) according to the manufacturer’s instructions. The 20 μL ddPCR reaction contained 10 μ L of 2x Supermix, 2 μL of gDNA, forward and reverse primer (each 0.4 μL), probe (0.2 μL), and 7 μL of nuclease-free water. We performed thermal cycling at 95°C for 10 min (denaturation), followed by 40 cycles, each at 94°C for 30 s and 56°C for 1 min, and a final 10 min incubation at 98°C. The ramp rate was 2°C/s. The distilled water was used as the template for the negative control. We analyzed each assay in four reactions in triplicate runs for the linearity analysis.

The annealing temperature and primer/probe concentrations of the ddPCR protocol were optimized. And the thermal gradient was optimized ranging from 54, 56, 58, 60, and 62°C on a Veriti™ 96-Well Thermal Cycler (Applied Biosystems, USA) using the gDNA of cultured *V. dahliae* and *V. longisporum*. The primer/probe concentrations ranged from 600 nM/400 nM, 500 nM/250 nM and 400 nM/100 nM were optimized with the identical annealing temperature at 56°C.

### Determination of limit of quantification (LoQ), limit of detection at 95% probability (LoD95%), and precision of measurement

The gDNA extracted from cultured *V. dahliae* and *V. longisporum* strains was used to determine the LoQ, LoD_95%,_ and measurement precision of each assay.

The LoQ of the qPCR and ddPCR is the lowest template concentration that an assay can accurately quantify based on the linearity of the standard curve. The relative standard deviation (RSD) of the LoQ value should be ≤25% (Kralik and Ricchi, 2017). To obtain the LoQ of the ddPCR and qPCR assays, we tested a series of 2-fold dilutions of *V. dahliae* and *V. longisporum* DNA, ranging from 1.2 to 296 copies/reaction (*V. dahliae*) and 1.6 to 400 copies/reaction (*V. longisporum*). Three independent experiments were done over two consecutive days, with 10 replicates of each concentration on each day.

The LoD_95%_ for ddPCR and qPCR is the lowest concentration at which the probability of detecting the target is 95% (Burns and Valdivia, 2008; Kralik and Ricchi, 2017). To determine the LoD_95%_ of both ddPCR and qPCR, we used the series of two-fold dilutions of gDNA at concentrations close to the detection limits as templates in each assay. We then assessed the LoD_95%_ using a probit analysis of the assay results, with the target concentration (dilution level) as an explanatory variable and the detection of the sample (positive/total) as a response variable. Three independent experiments were done over two consecutive days, with 10 replicates of each concentration on each day. The probit analysis with 95% confidence intervals (95% CI) was done using SPSS 17.0 software (Chicago, IL, USA).

The measurement precision was evaluated by calculating the RSD of the measured value at each dilution level (Kralik and Ricchi, 2017). For each concentration, we did three independent experiments over two consecutive days, with 10 replicates of each concentration on each day.

### ddPCR and qPCR inhibition by residual matrix

The inhibitory effect of the residual matrix on both ddPCR and qPCR assays was estimated by quantifying a constant amount of gDNA in the presence of different quantities (2 to 6 μL) of cotton root and soil extracts. We spiked the reactions with the same amount of gDNA of the two *Verticillium* species and evaluated the influence of extracts relative to the measured value for each sample without inhibitors. A Student's *t*-test was applied to evaluate the significance level of the ddPCR and qPCR assays. The preparation process was described by Maheshwari et al. (Maheshwari et al., 2017).

## Results

### Screening of primer/probe sets and phylogenetic analysis

To ensure the accurate classification of strains, we tested the 12 known isolates of fungi (*V. dahliae*, *V. longisporum*, *V. nonalfalfae*, *V. alboatrum*, *V. nigrescens*, *M. oryzae*, *B. maydis*, *E. turcicum*, *R. cerealis*, *M. incognita*, *F. pseudograminearum*, and Foc) and six known isolates of bacteria (*U. virens*, *A. citrulli*, Xoo, *P. syringae*, *R. solanacearum*, and Xcc) and aligned the respective ITS and 16s rRNA sequences. The results indicated that the classifications were correct.

To screen the optimum primer/probe set for high detection sensitivity, we performed qPCR to evaluate the five published primer/probe sets (**Table 1**) to detect *V. dahliae* and *V. longisporum*, using high-quality gDNA extracts from known cultures as templates. The results showed that the Cq values for the qPCRs varied between these sets for quantifying identical amounts of templates, and we obtained the lowest Cq values for both pathogens using the Vdl-1 primer/probe set (**Table S1**), which targets a multiple-copy rDNA IGS. According to subsequent sequencing, the amplification products confirmed that the Vdl-1 set amplified the 159-bp sequences of the *V. dahliae* and *V. longisporum* IGS sequences. The sequences were fairly similar and differed by one nucleotide from the NCBI published sequences (AF104926) in the alignments (**Figure S1**).

**Figure 1:**
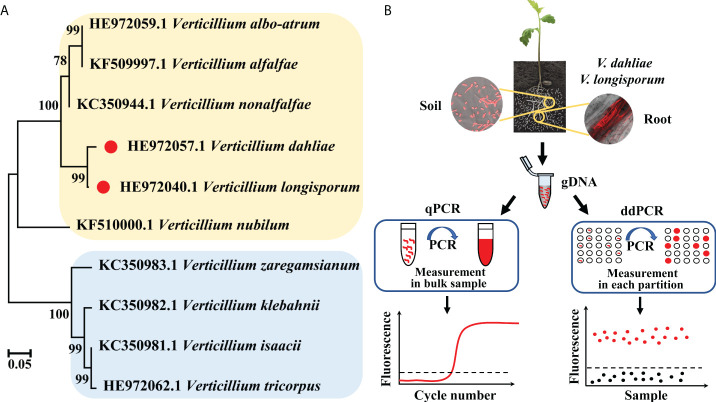
**(A)** Phylogenetic tree of *V. dahliae*, *V. longisporum*, and other *Verticillium* fungi based on IGS sequences. **(B)** Schematic diagram of the qPCR and ddPCR assays used for quantitative detection of *V. dahliae* and *V. longisporum*.

The phylogenetic tree based on the IGS sequences of major *Verticillium* fungi showed that they wereseparated into two distinct clades, with *V. dahliae* and *V. longisporum* having the closest relationship (**Figure 1A**). As soilborne pathogenic fungi, the spores of *V. dahliae* and *V. longisporum* exist in the soil and invade host plants through the roots, resulting in the typical symptoms of *Verticillium* infection. We detected the IGS fragment from the two fungi *via* a self-developed ddPCR workflow and, in parallel, a standard qPCR method, targeting the same IGS fragment. We compared the two methods, qPCR and ddPCR, based on different quantification principles (**Figure 1B**).

### qPCR assay development

**Figure 2:**
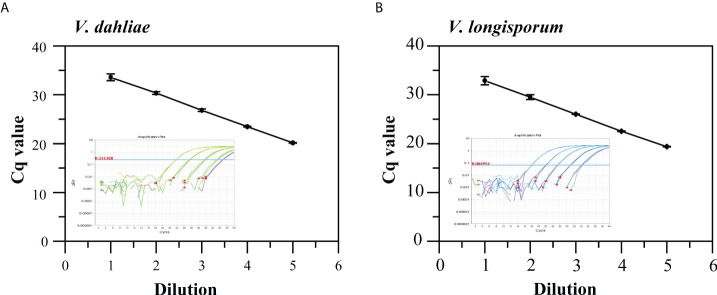
Standard curves and amplification curves of qPCR assays targeting IGS to detect *V. dahliae* **(A)** and *V. longisporum* **(B)**. Means of three independent measurements are shown; each error bar represents the standard deviation of the Cq values.

The standard curves generated from 10-fold serially diluted DNA of *V. dahliae* and *V. longisporum*, ranging from 2.23 ×10^-5^ to 2.23 × 10^-1^ and 4.61× 10^-5^ to 4.61 × 10^-1^ ng/μL, respectively, and used to validate qPCR using the Vdl-1 primer/probe set. As shown in **Figures 2A, B**, the qPCR assays had good linearity with each dilution level for both *V. dahliae* and *V. longisporum* gDNA (*R* ^2^ = 0.991 and 0.993, respectively). The scopes were -3.37 and -3.40, equivalent to a PCR efficiency of 97.9% and 96.8% for *V. dahliae* and *V. longisporum*, respectively. These results demonstrated the high-quality performance of the qPCR assay for detecting the two fungi based on the Vdl-1 primer/probe set. The results of the specificity evaluation of the qPCR assay showed that the runs were specific for *V. dahliae* and *V. longisporum* only, with no cross-reactivity with the other 10 fungal or 6 bacterial strains tested (**Table S2**).

### ddPCR assay development

**Figure 3:**
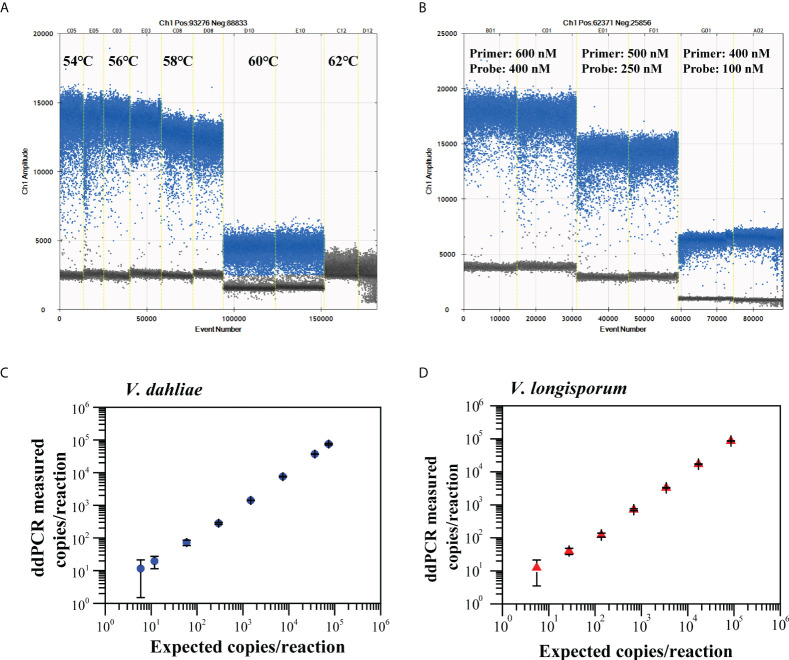
Optimization of annealing temperatures **(A)** and primer/probe concentrations **(B)** for ddPCR to quantify *V. dahliae* and *V. longisporum*. Linear regression of ddPCR assays for *V.* dahliae **(C)** and *V. longisporum* **(D)**. Means and standard deviation for each dilution series (*n* = 3) are shown.

The ddPCR assay for the quantitative detection of *V. dahliae and V. longisporum* using the validated Vdl-1 primer/probe set were tested at five annealing temperatures (54 to 62°C) (**Figures 3A**, **S2A**), then using various concentrations of primer/probe set (600/400, 500/250, and 400/100 nM) (**Figures 3B**, **S2B**). Based on the high resolution (well-separated positive and negative droplets) and low abundance of rain (droplets falling between the positive and negative populations), we selected an annealing temperature of 56°C and a primer/probe concentration of 500/250 nM for the subsequent ddPCR reaction.

We constructed the linear regression curve by plotting the log10-transformed copy number concentrations measured by ddPCR against the expected log10-transformed values of the serially diluted gDNA. The ddPCR assay showed good linearity for the quantification of *V. dahliae* and *V. longisporum* (*R* ^2^ = 0.998, 0.96 < slope < 1.03 and *R* ^2^ = 0.996, 0.95 < slope < 1.04, respectively) between the measured targets and the expected concentration values over a range of four orders of magnitude (**Figures 3C, D**). The corresponding LoQ was 37 copies/reaction for *V. dahliae* and 50 copies/reaction for *V. longisporum*, which met the criterion for the LoQ with a RSD lower than 25%. The results of the specificity evaluation showed that the optimized ddPCR assay was specific for *V. dahliae* and *V. longisporum* only, with no cross-reactivity with the other 16 similar pathogens tested (**Figure S3**).

### LoD_95%_, LoQ, and measurement precision

**Figure 4:**
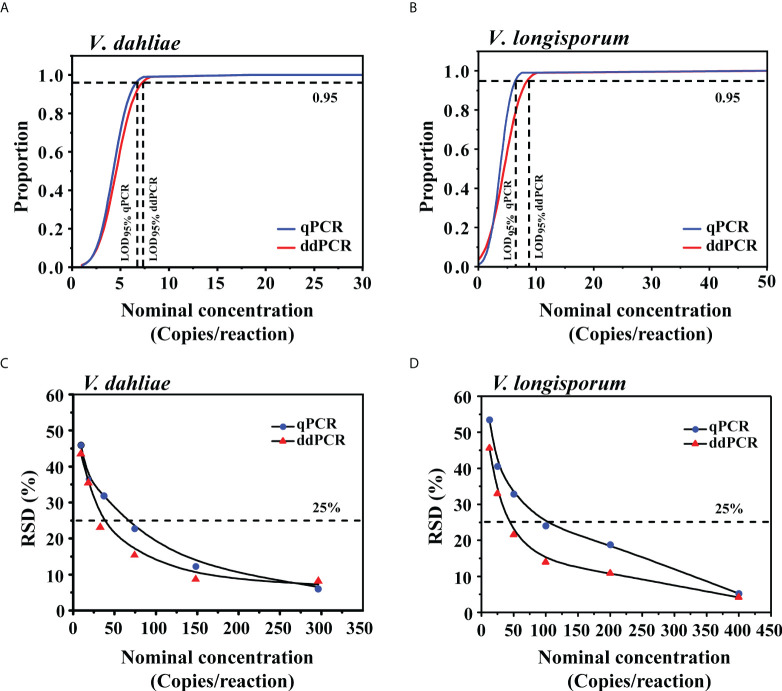
Comparison of LoD_95%_ and measurement precision for the qPCR and ddPCR to quantify *V. dahliae* and *V. longisporum*. **(A, B)** Determination of LoD_95%_ of qPCR and ddPCR by probit analysis. The *y*-axis shows the fraction of positive results in 10 parallel reactions for each given concentration indicated on the *x*-axis. Horizontal dashed lines indicate the dilution level where the estimated probability of detection is 95% (LoD_95%_). **(C, D)** RSD of qPCR and ddPCR at each low-concentration dilution level. The horizontal dashed lines indicate RSD = 25%.

To determine the LoD_95%_ of the ddPCR and qPCR assays, we used serial dilutions of gDNA, from 1.2 to 148 copies/reaction (*V. dahliae*) and 1.6 to 200 copies/reaction (*V. longisporum*), as templates in each assay. For *V. dahliae*, the probit analysis revealed a LoD_95%_ of 7.1 (95% CI: 5.5-14.3) copies/reaction for the ddPCR and 6.5 (95% CI: 5.2-12.1) copies/reaction for the qPCR in detecting *V. dahliae* (**Figure 4A**). For *V. longisporum*, the resulting LoD_95%_ was 8.5 (95% CI: 6.5-16.3) for the ddPCR and 6.4 (95% CI: 5.1-10.2) copies/reaction for the qPCR (**Figure 4B**). The above analysis showed that, with the same primer/probe set and templates, the sensitivities of the two methods for the identification of *V. dahliae* were similar, whereas qPCR was slightly more sensitive than ddPCR for detecting *V. longisporum* with a 95% probability.

To further compare the LoQ values of ddPCR and qPCR, we prepared a series of 2-fold DNA dilutions at concentrations close to the quantification limits. As shown in **Table S3**, the LoQ of qPCR was at least 74 and 100 copies/reaction for *V. dahliae* and *V. longisporum*, respectively, which were two times higher than those of the ddPCR assay. We investigated the measurement precision by calculating the RSD of the measured concentrations at each dilution level. The variability of the ddPCR assay was lower, as reflected in the low RSDs in comparison with those of the qPCR assay (**Figures 4C, D**).

### Diagnostic performance of ddPCR and qPCR using field samples

In the diagnostic comparison of ddPCR versus qPCR, 100 cotton root and 100 soil samples from a cotton field that had potentially been infected for years, were tested in parallel using the two methods. As shown in **Table 2**, the ddPCR had higher detection sensitivity than the qPCR for the two fungi. All the qPCR-positive cotton root and soil samples were also identified by ddPCR. Compared with the 48% and 30% positive rates for cotton root and soil samples, respectively, by qPCR, ddPCR improved the respective rates to 65% and 51%.

**Table 2:** Performance of qPCR and ddPCR assays using Vdl-1 primers to detect *V. dahliae* and *V. longisporum* in DNA extracted from cotton root and soil samples.

Sample	Analysis	Positive/Total
Cotton root	qPCR	48/100
	ddPCR	65/100
Soil	qPCR	30/100
	ddPCR	51/100

**Figure 5:**
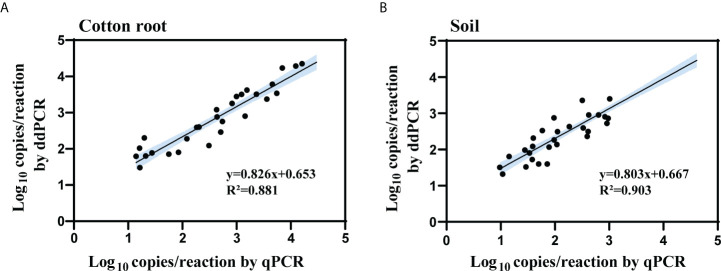
Linear regression and Pearson correlation analysis of measured copy number of the IGS target between qPCR and ddPCR for cotton root **(A)** and soil **(B)** samples. All cotton root and soil samples that tested positive by both methods were included in the analysis (cotton root samples, *n* = 48; soil samples, *n* = 30). *R* ^2^ is the proportion of variability in *y* that is attributable to *x*. Black solid line represents the correlation line; blue area represents the 95% confidence bands.

The samples tested double-positive in the ddPCR and qPCR were then used for linear regression and Pearson correlation analysis. The log copy number of IGS determined by ddPCR correlated well with that of qPCR for both types of collected samples (cotton root, R^2^ = 0.881, 0.735 < slope < 0.916; soil, R^2^ = 0.903, 0.727 < slope < 0.939) (**Figure 5**). For the 17 cotton root and 21 soil samples that tested negative by qPCR but positive by ddPCR, the average load was 26 and 38 copies/reaction in cotton root and soil samples, respectively.

**Figure 6:**
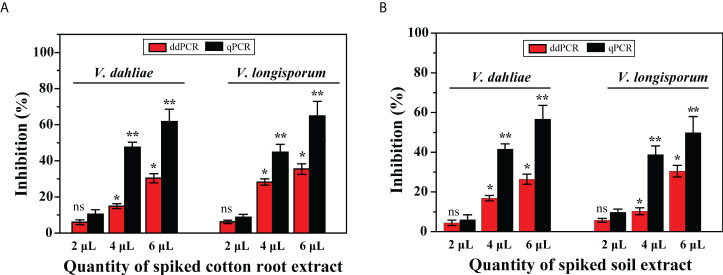
Effect of presence of DNA extracts from cotton roots and soil on quantification of *V. dahliae* and *V. longisporum* by ddPCR and qPCR assays. Samples were spiked with an equal amount of gDNA from both species and different quantities of **(A)** cotton root extracts and **(B)** soil extracts. Error bars represent standard error between three independent measurements of each run. Asterisks indicate statistically significant differences between treatments according to Student’s *t*-test. * and ** represent *P* ≤ 0.05 and *P* ≤ 0.01, respectively, compared with the inhibition control. ns, not significant.

Thus, compared with the sensitivity levels obtained with gDNA from cultures, the results showed that the positive diagnosis of the two pathogens using ddPCR was more robust than that using qPCR. To determine whether the residual matrix of field samples may cause the reduction in the amplification efficiency of qPCR, we prepared samples spiked with equal amounts of gDNA from cultured isolates (*V. dahliae*: 3.7 × 10^4^ IGS molecules; *V. longisporum*: 3.4 × 10^4^ IGS molecules) and different quantities of extracts from healthy cotton roots or distilled soil. The results showed that compared with the no-inhibition control (distilled water instead of extract), these extracts inhibited the quantification of the spiked DNA by both methods; with increasing extract amounts, less of the spiked DNA was measured by both methods. However, the resilience of ddPCR against such inhibition was higher than that of qPCR for quantifying *V. dahliae* and *V. longisporum* (**Figure 6**).

## Discussion

Digital PCR can directly and absolutely measure the copy number concentration of targets in a calibration-free manner with high sensitivity and precision, and shows “less susceptibility to PCR inhibitors” (Baker, 2012). To test the ability of digital PCR to quantitatively detect pathogens in samples, we evaluated qPCR and ddPCR in parallel to directly compare the analytical and diagnostic performance of the two methodologies for two destructive agents, *V. dahliae* and *V. longisporum*. Our findings indicated that the proposed ddPCR method is a more robust detection tool for pathogen detection, especially for infected field samples with low titers.

According to the Poisson distribution, where only sampling noise contributes to replicate variation, the LoD_95%_ of qPCR is three molecules (Burns and Valdivia, 2008; Kralik and Ricchi, 2017). In this study, the estimated LoD_95%_ of the qPCR assay (6.5 and 6.4 for *V. dahliae* and *V. longisporum*, respectively) was roughly close to the theoretical value, indicating that the qPCR assay was well-optimized. ddPCR did not appreciably improve the detection sensitivity of the two fungi when testing gDNA from pure cultures, and its LoD_95%_ values were even slightly higher than those of qPCR. However, the positive rates of ddPCR were substantially higher than those of qPCR, by 17% and 21% for the tested field cotton root and soil samples, respectively. Furthermore, the average target concentrations of the samples that tested negative by qPCR but positive by ddPCR were quite low, supporting the increased detection sensitivity of ddPCR for field samples. Although we did not further validate the positive samples determined by ddPCR using other methods, the results tended to be true positives, given the results of the specificity assessment of this assay. The results demonstrated that ddPCR allowed improved distinction of the presence of *V. dahliae* or *V. longisporum* in infected asymptomatic cotton samples, implying that it is able to detect the pathogens at an early stage of infection.

We further found that the presence of residual matrix from the extracts in the qPCR reaction, e.g., phenolic compounds and polysaccharides in cotton roots (Demeke and Adams, 1992; Osman and Rowhani, 2006), may substantially inhibit amplification efficiency and subsequently lead to underestimation of the pathogen titer of field samples or to the false negatives (Maheshwari et al., 2017). Aljawasim et al. discovered that several qPCR reactions for detecting *V. dahliae* were sensitive to PCR inhibitors, whereas others were not. They considered that for inhibitor-tolerant qPCR assays, the optimized DNA extraction protocol and the high concentration of tested gDNA may eliminate or minimize the influence of the PCR inhibitors in woody samples (Aljawasim and Vincelli, 2015). In contrast, as an end-point approach, ddPCR is less affected by variations in PCR efficiency (Hindson et al., 2013; Huggett et al., 2015); thus, we speculated that ddPCR is probably more resilient to differences in sample quality.

Additionally, the improved precision of ddPCR over qPCR in some applications has been reported (Hindson et al., 2013; Persson et al., 2018); therefore, it has been used to quantify reference materials for generating calibration curves of qPCR (Kline et al., 2009; Wang et al., 2021). The calibration curve required for qPCR is a major source of precision error, because consistent PCR efficiency is difficult to maintain, especially for low-level analytes (Alikian et al., 2017; Milosevic et al., 2018), and PCR efficiency for pure standards may differ from that of field samples in matrix composition (Corbisier et al., 2015). Here, the variability of the ddPCR assay quantitation of the two fungi was lower than those of qPCR at low pathogen densities, as reflected in lower standard errors; the LoQ performance of the ddPCR assay was also better than that of qPCR. The results of the inhibition analysis further showed that ddPCR was less affected by the matrix, underestimating quantities compared with qPCR, likely due to its partitioning of reactions into picoliter droplets, supporting the notion that PCR inhibitors potentially magnify quantification errors in qPCR systems (Hindson et al., 2013; Maheshwari et al., 2017).

Despite these advances, digital PCR has not yet been widely applied because it can be expensive, laborious, and time-consuming when not readily available (Huggett et al., 2013). Thus, the method may not now be suitable for large-scale screening of suspected samples, but it is a powerful complementary tool or may even be a better alternative for very low titer or poor quality samples. The combination of these two methods provides a more reliable pathogen-identification strategy to ensure the accuracy of the results.

This study was limited in that we assessed the performance of qPCR versus ddPCR assays using only one target. To reduce the potential systematic errors, assays for identifying more gene targets with different primer/probe sets need to be conducted in future studies. Although we focused on plant pathogens, our findings determined the empirical operating characteristics of qPCR and ddPCR, which should enable a broad variety of applications where sensitive and highly precise measurement is required.

## Conclusions

In this study to develop and validate a sensitive and accurate ddPCR assay to detect and quantify *V. dahliae* and *V. longisporum*, we found that ddPCR had higher measurement precision at low target concentrations, increased detection sensitivity for field samples, and less susceptibility to the effects of matrix inhibitors compared with qPCR. The ddPCR methodology that we established provided more accurate quantification of these destructive pathogens with a higher level of sensitivity, which will facilitate early intervention and improved outcomes for plant protection.

## Data availability statement

The original contributions presented in the study are included in the article/**Supplementary Material**. Further inquiries can be directed to the corresponding authors.

## Author contributions

Conceptualization, XS and YG. Methodology, DW and XJo. Software, SC. Data curation, HJ, SC, XJn, and YW. Writing-original draft preparation, review and editing, DW, YG and XS. Funding acquisition, DW, HJ and YW. All authors have read and agreed to the published version of the manuscript. All authors contributed to the article and approved the submitted version.

## Funding

This research was funded by grants from the National Major Special Project for the Development of Transgenic Organisms (2016ZX08012-003), the Quality and Basic Ability Construction Project of National Institute of Metrology, China (ANL2202), the Science and Technology Project of Guangxi (2020450000340001) and the Hebei Technology Innovation Center for Green Management of Soil-borne Diseases (Baoding University) (2021K08).

## Conflict of interest

The authors declare that the research was conducted in the absence of any commercial or financial relationships that could be construed as a potential conflict of interest.

## Publisher’s note

All claims expressed in this article are solely those of the authors and do not necessarily represent those of their affiliated organizations, or those of the publisher, the editors and the reviewers. Any product that may be evaluated in this article, or claim that may be made by its manufacturer, is not guaranteed or endorsed by the publisher.
