# Supplementary material for: Detection and quantification of Verticillium dahliae and V. longisporum by droplet digital PCR versus quantitative real-time PCR
Source: Front Cell Infect Microbiol. 2022 Aug 22;12:995705. doi: 10.3389/fcimb.2022.995705 (PMC9441566; doi:10.3389/fcimb.2022.995705)
Supplement: Supplementary file 1 [file DataSheet_1.docx]

Supplementary Materials


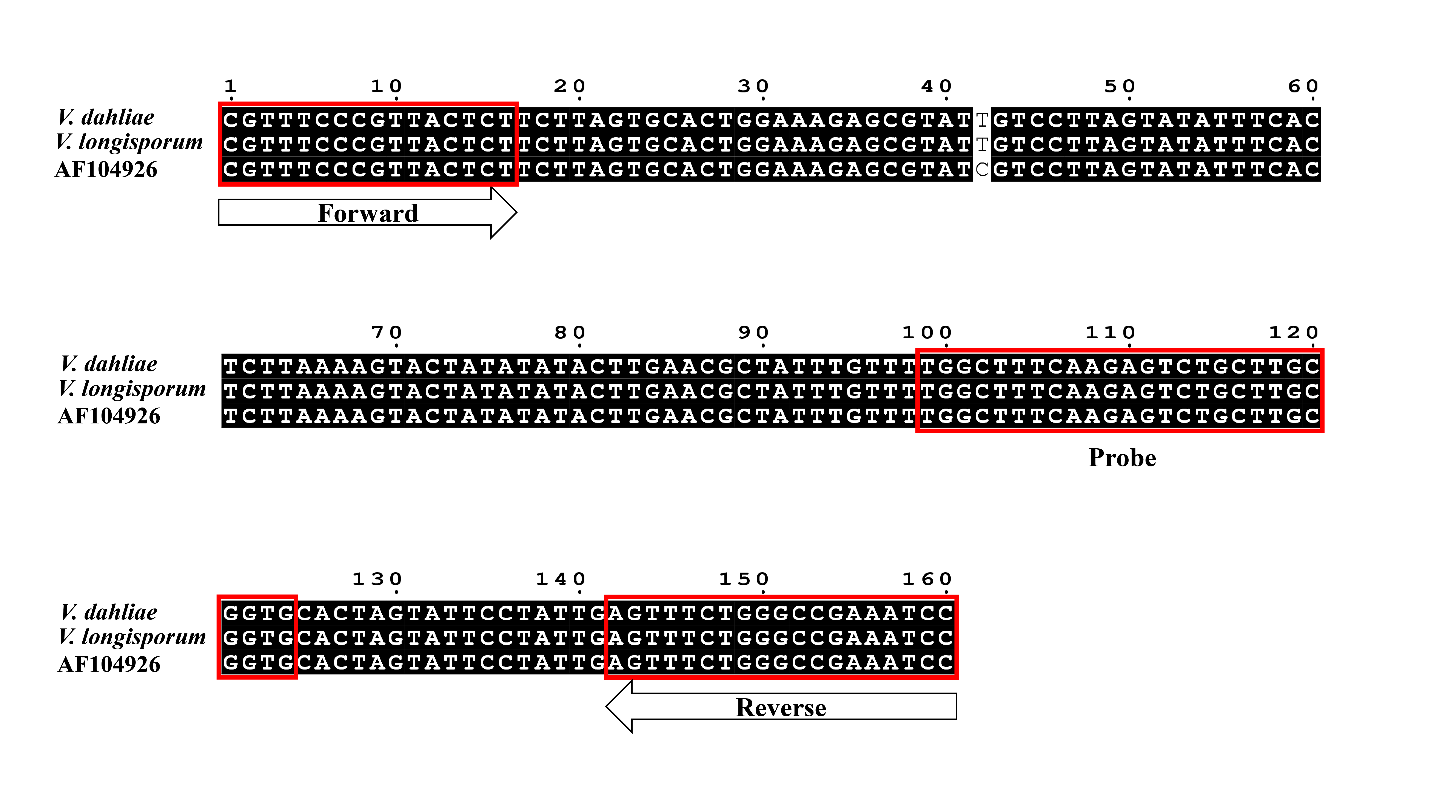


**Figure S1.** The amplified sequences of *V. dahliae* and *V. longisporum* by Vdl-1 prime/probe set.


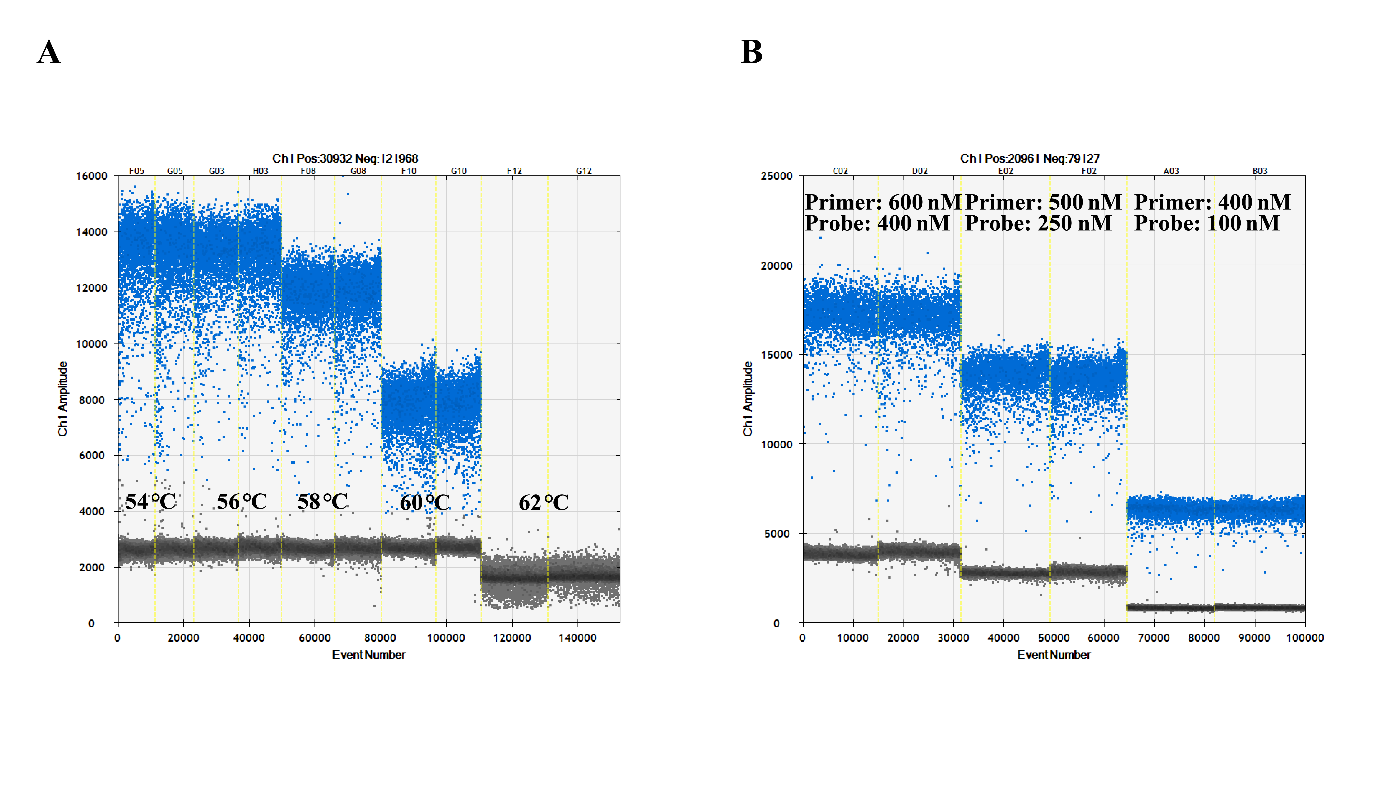


**Figure S2.** Ampliﬁcation plots from optimization experiments on ddPCR. (A) Gradient PCR for optimizing annealing temperature for detecting *V. longisporum*. (B) Different concentrations of primer/probe tested for optimizing appropriate concentrations used for detecting *V. longisporum*. In these plots, blue spots denate the positive droplets and grey spots denate the negative droplets without any target DNA.


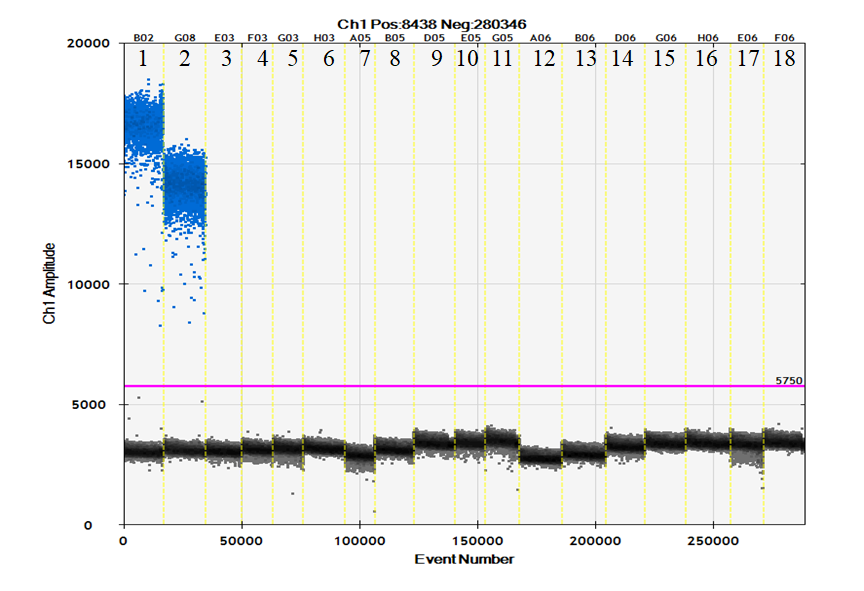


**Figure S3.** The specificity detection of ddPCR. 1: *V.* *dahliae*; 2: *V.* *longisporum*; 3: *V.* *nonalfalfae*; 4: *V.* *alboatrum*; 5: *V.* *nigrescens*; 6: *Magnaporthe* *oryzae*; 7: *Bipolaris* *maydis*; 8: *Exserohilum* *turcicum*; 9: *Fusarium* *oxysporum* f. sp. *conglutinans*; 10: *Rhizoctonia* *cerealis*; 11: *Meloidogyne* *incognita*; 12: *Fusarium* *pseudograminearum*; 13: *Ustilaginoidea* *virens*; 14: *Acidovorax* *citrulli*; 15: *Xanthomonas* *oryzae* pv. *oryzae*; 16: *Pseudomonas syringae*; 17: *Ralstonia* *solanacearum*; 18: *Xanthomonas* *campestris* pv.campestris. Pink line is the threshold.

**Table S1. Cq values of qPCR assays for detection of V. dahliae and V. longisporum by different primer/probe sets.**

| **fungus DNA** | **Concentration（ng/μL）** | **Vdl-1** | **Vdl-2** | **Vdl-3** | **Vdl-4** | **Vdl-5** |
| --- | --- | --- | --- | --- | --- | --- |
| *V. dahliae* | 2.1 | 16.05 | 22.47 | 26.83 | 23.63 | 16.65 |
| *V. longisporum* | 8.0 | 14.42 | 17.45 | 27.15 | 22.64 | 14.96 |

**Table S2.** Results of specificity analysis using Vdl-1 primer/probe set.

| **Fungus** | **Cq value** |
| --- | --- |
| *V. nonalfalfae* | Un. |
| *V. alboatrum* | Un. |
| *V. nigrescens* | Un. |
| *Magnaporthe oryzae* | Un. |
| *Bipolaris maydis* | Un. |
| *Exserohilum turcicum* | Un. |
| *Fusarium oxysporum f. sp. conglutinans* | Un. |
| *Rhizoctonia cerealis* | Un. |
| *Meloidogyne incognita* | Un. |
| *Fusarium pseudograminearum* | Un. |
| *Ustilaginoidea virens* | Un. |
| *Acidovorax citrulli* | Un. |
| *Xanthomonas oryzae pv. oryzae* | Un. |
| *Pseudomonas syringae* | Un. |
| *Ralstonia solanacearum* | Un. |
| *Xanthomonas campestris pv.campestris* | Un. |

**Table S3.** Copy number concentration of IGS in serial dilution of *V. dahliae* and *V. longisporum* DNA determined by gravimetrical value and ddPCR (cp/r, copies/reaction).

| **Dilution** | | **Input (cp/r)** | **Measured by ddPCR**  **(cp/r)** | | **ddPCRRSD**  **(%)** | | **Measured by qPCR**  **(cp/r)** | | | **qPCR RSD**  **(%)** | |
| --- | --- | --- | --- | --- | --- | --- | --- | --- | --- | --- | --- |
| *V. dahliae* | S5 | 296.4 | | 280.0 | | 7.2 | | 275.0 | 8.2 | |  |
|  | S6 | 148.2 | | 155.0 | | 10.2 | | 157.0 | 12.2 | |  |
|  | S7 | 74.1 | | 85.0 | | 16.5 | | 68.0 | 22.7 | |  |
|  | S8 | 37.1 | | 45.0 | | 23.7 | | 21.0 | 31.9 | |  |
| *V. longisporum* | A5 | 400.0 | | 416.0 | | 5.2 | | 412.0 | 5.2 | |  |
|  | A6 | 200.0 | | 189.0 | | 11.8 | | 184.0 | 11.8 | |  |
|  | A7 | 100.0 | | 110.0 | | 13.9 | | 89.0 | 24.0 | |  |
|  | A8 | 50.0 | | 58.0 | | 19.5 | | 31.0 | 32.9 | |  |
